# AAPM Medical Physics Practice Guideline 2.a: Commissioning and quality assurance of X‐ray‐based image‐guided radiotherapy systems

**DOI:** 10.1120/jacmp.v15i1.4528

**Published:** 2014-01-06

**Authors:** Jonas D. Fontenot, Hassaan Alkhatib, Jeffrey A. Garrett, Andrew R Jensen, Steven P. McCullough, Arthur J. Olch, Brent C. Parker, Ching‐Chong Jack Yang, Lynne A. Fairobent

**Affiliations:** ^1^ AAPM Staff

## Abstract

The American Association of Physicists in Medicine (AAPM) is a nonprofit professional society whose primary purposes are to advance the science, education, and professional practice of medical physics. The AAPM has more than 8,000 members and is the principal organization of medical physicists in the United States.

The AAPM will periodically define new practice guidelines for medical physics practice to help advance the science of medical physics and to improve the quality of service to patients throughout the United States. Existing medical physics practice guidelines will be reviewed for the purpose of revision or renewal, as appropriate, on their fifth anniversary or sooner.

Each medical physics practice guideline represents a policy statement by the AAPM, has undergone a thorough consensus process in which it has been subjected to extensive review, and requires the approval of the Professional Council. The medical physics practice guidelines recognize that the safe and effective use of diagnostic and therapeutic radiology requires specific training, skills, and techniques, as described in each document. Reproduction or modification of the published practice guidelines and technical standards by those entities not providing these services is not authorized.

## 1. Introduction

Image‐guided radiation therapy (IGRT), in its many forms, is an important tool in improving the effectiveness of clinical radiation oncology. IGRT involves the use of patient images to localize and reposition the patient or delivery system prior to treatment to ensure that the therapeutic beam is correctly directed toward the target. IGRT imaging strategies have utilized X‐rays, ultrasound, and other means. In particular, IGRT has been most commonly facilitated using X‐rays, beginning with use of megavoltage (MV) portal and/or orthogonal setup images some decades ago. These images provide a means of evaluating the position of the treatment isocenter and field edges relative to the patient position. Because of the poor low‐contrast resolution of MV images, bony anatomy may be taken as a surrogate of the target volume, which is frequently soft‐tissue‐equivalent and not clearly visible within the image. However, as many studies have shown, the target volume can exhibit a different relative location to bony anatomy than expected. One solution to clinical scenarios in which improved soft‐tissue targeting is desired was the introduction of in‐room kilovoltage (kV) imaging systems. Such systems have included computed tomography (CT) scanners located within the treatment room (e.g., “CT‐on‐rails”) and kV imaging systems affixed to the floor/ceiling or to the linear accelerator gantry itself. These systems have provided improved low‐contrast localization of soft‐tissue targets and — in the case of in‐room CT scanners and gantry‐mounted imaging systems — allowed for acquisition of pretreatment volumetric images.

The specific choice and application of IGRT strategy depends on the complexity and requirements of the treatment in question. It may serve as an enhancement to an established technique (as in the case of three‐dimensional conformal radiation therapy or intensity‐modulated radiation therapy) or as a necessary and critical component of the treatment process (as in the case of stereotactic body radiation therapy^(1)^). Currently, IGRT strategies are being used more than ever before, and various forms of IGRT have been, are, and will continue to be important tools in radiation therapy. The report of AAPM Task Group 104^(2)^ provides an instructive overview the various uses of X‐ray imaging in radiation therapy.

As the clinical treatment process continues to rely more heavily on IGRT strategies, the Qualified Medical Physicist (QMP) is under increasing pressure to maintain patient safety and treatment quality through quality assurance programs that are in step with the rapid pace of technological development. Many clinical practice environments now utilize treatment delivery systems with one or more IGRT systems that fall under the responsibility of the QMP. A variety of guidance documents and task groups reports have been issued that include additional recommendations for commissioning and quality assurance of IGRT systems.^2^, ^3^, ^4^, ^5^, ^6^, ^7^, ^8^, ^9^ However, these reports do not clearly delineate *best* practice from *minimum* practice standards.

### a. Goals and Rationale

This document is part of a series of medical physics practice guidelines commissioned by the American Association of Physicists in Medicine (AAPM) intended to succinctly state the minimum acceptable standards for various aspects of clinical medical physics. While implementation of robust and comprehensive quality assurance programs recommended in other reports from the AAPM is encouraged, the purpose of this particular report is to describe the minimum acceptable practice standards for the commissioning and quality assurance of X‐ray‐based image‐guidance systems utilized in radiotherapy. This document is not intended to replace or revise previous AAPM Task Group Reports, but to assist the QMP in establishing and maintaining a safe and effective IGRT program by providing an overview of the minimum requirements and needs of X‐ray‐based IGRT systems. Indeed, the reader is referred to the appropriate technical reference documents or task group reports in instances when additional recommendations beyond minimum practice guidelines are desired.^1^, ^2^, ^3^, ^4^, ^5^, ^6^, ^7^, ^8^ Finally, the standards and procedures described in this document are applicable to the imaging guidance system insofar as its resulting images are used to position the patient and/or localize the target volume. Use of IGRT system hardware and software for other purposes, such as dose calculation, are beyond the scope of this report. Technologies covered by these guidelines include:

- Gantry‐mounted two‐dimensional MV imaging systems
- Gantry‐mounted three‐dimensional MV imaging systems
- Gantry‐mounted two‐dimensional kV imaging systems
- Gantry‐mounted three‐dimensional kV imaging systems
- Room‐mounted two‐dimensional kV imaging systems
- Room‐mounted three‐dimensional kV imaging systems

In this context, “gantry‐mounted” imaging systems are those in which the mechanical movement of the imaging hardware is coupled with mechanical movement of the treatment delivery device (e.g., Varian/Elekta/Siemens on‐board kV imaging systems, electronic portal MV imaging systems, TomoTherapy megavoltage CT, etc.). “Room‐mounted” systems include all imaging systems not coupled with the treatment delivery device (e.g., ExacTrac, CyberKnife, in‐room CT, etc.). Fluoroscopy modes are also within the scope of this report.

### b. Intended Users

The intended users of this report are QMPs who seek to understand the technical requirements of clinical implementation and quality assurance of a safe IGRT practice, and administrators interested in the resources required for IGRT.

## 2. Definitions and Abbreviations

- CBCT ‐ cone‐beam computed tomography
- IGRT ‐ image‐guided radiotherapy
- kV ‐ kilovoltage
- MV ‐ megavoltage
- OIS ‐ oncology information system
- QA ‐ quality assurance
- TPS ‐ treatment planning system

## 3. Staff Qualifications and Responsibilities

Implementation of a successful IGRT program requires contributions from each member of the treatment team. Recommendations for staff qualifications and responsibilities are consistent with those described by the ACR‐ASTRO practice guideline for clinical use of IGRT.^(9)^

a. Medical Physicist — The qualified medical physicist (QMP) **must** be competent to practice independently in the subfield of therapeutic radiological physics. The individual **must** be certified either by the American Board of Radiology, American Board of Medical Physicists, or the Canadian College of Medical Physicists. Responsibilities of the qualified medical physicist in an IGRT program include:

- Acceptance testing and commissioning
- Implementing and managing a quality assurance program
- Developing and implementing standard operating procedures (including imaging protocols and repositioning thresholds)

The QMP may be assisted by medical physics residents or medical physicist assistants with these responsibilities provided 1) these individuals have been appropriately trained to perform the assigned tasks, and 2) the QMP provides general supervision of all work performed.

b. Radiation Oncologist — The radiation oncologist **should** meet qualifications outlined in the ACR‐ASTRO practice guideline for clinical use of IGRT.^(10)^ In short, the responsibilities of the radiation oncologist in an IGRT program include:

- Specifying patient positioning procedures
- Specifying imaging modalities and frequencies
- Identifying registration targets and repositioning thresholds
- Conducting timely review of clinical IGRT images
- Conducting regular reviews of the IGRT program
- Implementing and managing a quality assurance program
- Developing and implementing standard operating procedures (including imaging protocols and repositioning thresholds)

c. Medical Dosimetrist — The medical dosimetrist **should** meet the qualifications outlined in the *Scope of Practice of a Medical Dosimetrist* approved by the Board of Directors of the American Association of Medical Dosimetrists.^(11)^ Responsibilities of the medical dosimetrist or treatment planner in an IGRT program include:

- Creating and transferring to the OIS all patient‐specific data necessary for IGRT implementation

d. Radiation Therapist — The radiation therapist should meet the qualifications outlined in *Radiation Therapy Practice Standards* issued by the American Society of Radiologic Technologists.^(12)^) Responsibilities of the radiation therapist in an IGRT program include:

- Understanding the use of positioning devices in IGRT
- Preparing the IGRT system for acquisition of patient‐specific positioning verification images
- Implementing the IGRT treatment protocol under the supervision of the radiation oncologist and medical physicist
- Acquiring positioning verification images for review by the radiation oncologist
- Assisting in periodic review of the stability of the IGRT system (e.g., daily QA)

e. Information Technologist — It is important that each facility identify an individual that is responsible for providing and maintaining resources necessary for storing, archiving and retrieving images generated during IGRT. This may be accomplished by a dedicated Information Specialist or duties assigned to another team member.

## 4. Implementation Guidelines

### a. Minimum required resources and equipment

#### i. Staffing

Approximate time requirements needed for implementation, maintenance and quality assurance of each IGRT program type (per each IGRT system) are provided below. Estimates are provided as general reference values only, and are not intended to justify site‐specific staffing models or physics time for specific billing codes. “Acceptance/commissioning” includes all activities needed for IGRT program implementation, including documentation. “Documentation” refers to creation of a formal commissioning report, and drafting of policies and procedures specific to clinical use and routine quality assurance of IGRT (including creating QA forms and templates). “Ongoing support” includes all activities needed for maintenance of an established IGRT program (e.g., routine quality assurance, troubleshooting, upgrades, service/repairs).

Two‐dimensional MV imaging systems
  - Acceptance/Commissioning/Documentation: 18‐36 hours
  - Ongoing support: 25‐50 hours annually
2. Two‐dimensional kV imaging systems
  - Acceptance/Commissioning/Documentation: 18‐36 hours
  - Ongoing support: 25‐50 hours annually
3. Three‐dimensional MV imaging systems
  - Acceptance/Commissioning/Documentation: 18‐36 hours
  - Ongoing support: 100‐125 hours annually
4. Three‐dimensional kV imaging systems
  - Acceptance/Commissioning/Documentation: 18‐36 hours
  - Ongoing support: 100‐125 hours annually

#### ii. Equipment

Quality assurance phantoms and tools must provide reliable values of the measured parameters and can be used to judge whether tolerance criteria have been achieved. In many cases, manufacturers of IGRT systems provide quality assurance phantoms which can be used for quality assurance purposes. In‐house and commercial phantoms specifically designed for IGRT are also available and, when coupled with automated image analysis tools, may improve efficiency. At a minimum, quality assurance tools **must** be capable of assessing the following IGRT characteristics:

- Image quality
- Spatial accuracy (scaling)
- Congruence of imaging and treatment isocenters
- Accuracy of registration/couch movements
- Imaging dose

### b. Staff training

Training for the operation of the IGRT system **must** be provided. The IGRT system vendor typically provides on‐site training to the physicist and therapists for use of the equipment. Prior to initial use of IGRT, the treatment team **should** meet to discuss staff responsibilities, clinical goals, and process workflows. The physicist **should** also review the image acquisition procedures with the therapists and radiation oncologists. In general, IGRT training will require additional dedicated staff time that is not included in the estimated time requirements of Section 4.a.1.

### c. Process descriptions

Example procedures for each of the tests recommended in Table 1 are described below. The approximate time needed to complete each procedure is noted in parenthesis following the process description. In some cases, customer acceptance procedures provided by the equipment vendor satisfactorily meet the stated practice standards; however, it is the responsibility of the QMP to judge the adequacy and completeness of all measurements needed for use of a particular IGRT system. It is important to note that these are only example procedures, and a variety of methods may be used to complete the recommended tests. Certain commercially available products are referred to by name. These references are for informational purposes only, and imply neither endorsement by the AAPM nor that these are the best or the only products available for the stated purpose.

#### i. Customer acceptance procedures (all systems)

The QMP **must** provide direct supervision during the acceptance testing process.^(13)^ Customer acceptance tests procedures are intended to ensure that the imaging equipment satisfies the performance requirements stated in the purchase agreement. In some cases, measurements completed as part of the acceptance procedures may also serve as components in establishing the routine quality assurance program. The vendor **must** demonstrate acceptable system performance. (Time: 8‐16 hours)

#### ii. TPS configuration and connectivity (2D systems)

Digitally reconstructed radiographs (DRR) of test objects in various orientations are created with the treatment planning system and transferred (typically via DICOM interface) to the image guidance system. Proper display of the DRR image within the image‐guidance software **must** be ensured. (Time: 3‐4 hours)

#### iii. TPS configuration and connectivity (3D systems)

Reference CT image sets of test objects in various orientations are imported into the treatment planning system. Contours are added and the images and structures are transferred (typically via DICOM interface) to the image‐guidance system. Proper display of the reference CT images and structures within the image‐guidance software **must** be ensured. (Time: 3‐4 hours)

#### iv. OIS integration and connectivity (2D systems)

Setup fields created for a test patient within the oncology information system are properly recognized by the imaging hardware and software when loaded. Acquired images are then assigned to the correct patient, if applicable. (Time: 2‐3 hours)

**Table 1:** Recommended minimum practices for commissioning and QA of an IGRT system

*Acceptance Testing and Commissioning*	
*Procedure*	
Customer acceptance procedures	
TPS integration	
OIS integration	
Establish routine QA baselines	
QA documentation	
*Routine Quality Assurance*	
*Procedure*	*Tolerance*
*Daily*	
*Safety/interlocks*	*Functional*
Imaging‐treatment isocenter coincidence (SRS only)	1 mm
Positioning/repositioning (SRS only)	1 mm
Imaging‐treatment isocenter coincidence (SBRT only)	2 mm
Positioning/repositioning (SBRT only)	2 mm
*Weekly*	
Imaging‐treatment isocenter coincidence (non‐SRS/SBRT)	2 mm
Positioning/repositioning (non‐SRS/SBRT)	2 mm
*Semi‐annually*	
Image scaling	2 mm
*Annually*	
Imaging dose	
2D MV	±1cGy of baseline value
2D kV (static imaging mode)	±3mGy of baseline value
2D kV (fluoroscopy mode)	±1cGy/min of baseline value
All 3D imaging modes	±1cGy of baseline value
Image quality	
2D (spatial resolution, contrast)	Baseline value
3D (uniformity, spatial resolution, contrast)	
*Upgrade/Repair/Service*	
Verify / Reestablish QA baselines (as appropriate)	

a SRS=stereotactic radiosurgery; SBRT=stereotactic body radiation therapy.

#### v. OIS integration and connectivity (3D systems)

Volumetric IGRT image fields (CBCT, MVCT, CT‐on‐rails) created for a test patient within the oncology information system are properly loaded and recognized by the imaging hardware and software. Acquired images are assigned to the correct patient, if applicable. (Time: 2‐3 hours)

#### vi. Routine QA baselines (all systems)

Measurements taken at the time of IGRT system commissioning which characterize IGRT system performance will serve as reference values for the routine QA program. See Table 1 for recommended QA tests requiring reference measurements. (Time: 2‐3 hours)

#### vii. IGRT QA program documentation (all systems)

All acceptance and commissioning procedures and results **must** be contained within a formal report. Furthermore, a formal policy for routine IGRT QA program and procedures for performing routine QA measurements **must** be developed. (Time: 4‐8 hours)

#### v. Safety/interlocks (all systems)

With image acquisition initiated, ensure beam termination occurs when the treatment room door is opened (if applicable) and when any termination keys are depressed. If images are to be acquired with the treatment room door open, then measurements and calculations of exposure should be performed at the treatment console to ensure safe operating conditions. Also ensure that gantry rotation is terminated when touch guards are depressed. Verify that indicator lamps are illuminated during image acquisition (Time: 5 minutes)

#### vi. Contrast (2D kV systems)

A phantom with low‐contrast objects (such as the Leeds phantom) is placed on the treatment couch at isocenter. A planar kV image is acquired using a reference technique determined at the time of acceptance testing. The window and level are adjusted to reference values determined at the time of acceptance testing. The number of visible disks is recorded, with more indicating better low‐contrast visibility. (Time: 15 minutes)

#### vii. Contrast (2D MV systems)

A phantom with low‐contrast objects (such as the Las Vegas phantom) is placed on the treatment couch. A planar MV image is acquired using a reference technique determined at the time of acceptance testing. The window and level are adjusted to a reference value determined at the time of acceptance testing. The number of visible disks of largest diameter or frequency groups is recorded, with more indicating better low‐contrast visibility. (Time: 15 minutes)

#### viii. Contrast (3D systems)

An appropriate volumetric image quality phantom (such as a CT phantom) is positioned on the treatment table using the room lasers. A volumetric image is acquired using a reference technique determined at the time of acceptance testing. Either the difference in CT number of different materials within the phantom, or the number of visible low‐contrast objects is recorded. (Time: 15 minutes)

#### ix. Spatial resolution (2D kV systems)

A phantom with high‐contrast objects (such as the Leeds phantom) is placed on the treatment couch. A planar kV image is acquired using a reference technique determined at the time of acceptance testing. The number of frequency groups that are clearly distinguished is recorded, with more frequency groups indicating better spatial resolution. (Time: 15 minutes)

#### x. Spatial resolution (2D MV systems)

A phantom with high‐contrast objects (such as the Las Vegas phantom) is placed on the treatment couch. A planar MV image is acquired using a reference technique determined at the time of acceptance testing. The number of visible disks of greatest contrast is recorded, with more disks indicating better spatial resolution. (Time: 15 minutes)

#### xi. Spatial resolution (3D systems)

An appropriate volumetric image quality phantom (such as the Catphan) is positioned on the treatment table using the room lasers. A volumetric image is acquired using a reference technique determined at the time of acceptance testing. The number of frequency groups that are clearly distinguished is recorded, with more frequency groups indicating better spatial resolution. (Time: 15 minutes)

#### xv. Scaling (all systems)

A phantom of known dimensions is placed on the treatment table using the room lasers. A planar or volumetric image is acquired. Window and level are adjusted such that phantom is clearly visible. The distance between two objects of known separation in the horizontal and vertical axes is recorded and compared with the known distance. For 3D imaging systems, scaling **must** be measured in all 3 dimensions. (Time: 10 minutes)

#### xvi. Uniformity (3D systems)

An appropriate volumetric image quality phantom (such as the Catphan) is positioned on the treatment table using the room lasers. A volumetric image is recorded. The average pixel value over a region of interest at multiple locations (e.g., center, 12 o'clock, and 3 o'clock) is recorded and compared. (Time: 15 minutes)

#### xvii. Imaging‐treatment isocenter coincidence (2D systems)

A variety of methods may be used to verify congruence of imaging and treatment isocenters. Most of these techniques require alignment of a radiopaque marker (e.g., ball bearing, fiducial, or commercial device) to treatment isocenter via room lasers. Orthogonal or oblique images are then acquired. Congruence of imaging and treatment isocenters is verified by comparing the position of the markers with the center of the orthogonal/oblique images. (Time: 10‐15 minutes)

#### xviii. Imaging‐treatment isocenter coincidence (3D systems)

A variety of methods may be used to verify congruence of imaging and treatment isocenters. Most of these techniques are usually coupled with the “positioning/repositioning” test and begin with positioning of a radiopaque marker (ball bearing, fiducial, or commercial device) on the treatment table with known offsets from isocenter. Volumetric IGRT images are then acquired and registered with reference images. The recommended couch shifts from the image registration software are recorded and applied, and coincidence of the imaging and treatment isocenters is assessed by 1) comparing the position of the marker with the room lasers, or 2) comparing the coincidence of the marker with the center position of an acquired MV portal image, if available. (Time: 10‐15 minutes)

#### xix. Positioning/Repositioning (all systems)

A radiopaque marker is positioned on the treatment table with known offsets from isocenter. Images are acquired and registered with reference images. The recommended couch shifts from the image registration software are recorded and applied. Proper functioning of the image registration software is verified by comparing the recommended couch shifts with the known offsets, while correct application of the shifts is assessed by comparing the position of the marker with the room lasers. (Time: 10‐15 minutes)

#### xx. Imaging dose (3D systems)

Several different methods are currently used to characterize the dose from 3D IGRT systems. The traditional metric for dose from CT imaging, the computed tomography dose index (CTDI), has been applied to IGRT imaging. More recently, the AAPM Task Group 111^(9)^ report has introduced a new metric, the cumulative dose. These two different metrics use different measurement equipment and irradiation geometries to characterize the dose.

Measurement equipment used to measure CTDI includes a calibrated 100 mm long pencil ionization chamber and an appropriate phantom, one simulating a head and the other a large pelvis. For each imaging mode, the phantom that matches the mode's target anatomy is used. The phantom is positioned at isocenter and the pencil chamber is centered in the phantom. The radiation field length **must** be constrained to be less than the active length of the pencil ionization chamber. For each imaging mode, measurements are repeated for the central and peripheral phantom locations.

Measurement equipment used to measure the cumulative imaging dose includes a Farmer‐type ionization chamber calibrated in the appropriate energy range and several acrylic CTDI phantoms. For each imaging mode, the phantoms that match the mode's target anatomy are used. The CTDI phantoms are abutted until their length exceeds the length of the radiation field. The phantom is centered at isocenter and the Farmer chamber is centered in the phantom. For each imaging mode, measurements are repeated for the central and peripheral phantom locations.

Measured imaging dose **must** be documented and its management **should** be approached with the goal of keeping it as low as necessary to achieve clinically useful images. (Time: 15‐60 minutes, depending on the number of techniques measured)

#### xxi. Imaging dose (2D systems)

Imaging dose from 2D kV systems is most typically characterized using entrance surface air kerma (skin exposure). Measurement equipment used to measure the entrance air kerma includes a calibrated ionization chamber and a phantom. The ionization chamber is placed between the source and the phantom in such a way as to minimize scatter radiation to the ionization chamber. The field size is set to cover the detector. A clinically relevant beam is delivered, and the air kerma rate is calculated for static and fluoroscopic imaging modes, respectively.

Measured imaging dose **should** be documented and its management **should** be approached with the goal of keeping it as low as necessary to achieve clinically useful images. (Time: 15‐60 minutes, depending on the number of techniques measured)

### d. Continuing quality improvement

Ongoing review and audit of the IGRT program **should** occur at regular intervals. In particular, periodic review of clinical image registration, the use of appropriate imaging techniques/frequencies, adherence to stated QA programs, and revision of IGRT strategies based on pertinent changes in clinical practices **should** be assessed.

## 5. Recommendations

Recommended minimum practices for commissioning and QA of an IGRT system are shown in Table 1. Test frequencies and tolerance values were developed based on relevant AAPM Task Group reports and the experience of the MPPG members in relation to the stability and importance of each parameter to the IGRT process. Sample process descriptions are included in Section 5.c. The “baseline value” shown in the table refers to the IGRT system manufacturer's minimum performance standard stated in the customer acceptance procedure documentation. If unavailable or not specified, then “baseline value” can be taken as the value measured at the time of commissioning. For example, most IGRT system manufacturers have stated performance specifications for image quality and, in such cases; those may serve as the tolerance values for routine QA measurements of image quality. However, most IGRT system manufacturers do not have stated performance specifications for imaging dose and, in such cases, the imaging dose measured at the time of commissioning may serve as the baseline value to which future measurements are compared.

In general, the frequency of routine QA tests is proportional to the importance of their performance for the purpose of patient alignment. As such, evaluation of imaging‐treatment isocenter coincidence and positioning/repositioning is considered critical. While daily checks of these parameters are preferred, weekly checks are considered acceptable for IGRT systems used with standard fractionation schemes. For IGRT systems used for SRS/SBRT, daily QA testing frequency **must** also be required on days when procedures are scheduled.

The imaging dose from an IGRT system **must** be measured for at least one acquisition technique of each mode of clinical operation. For example, a gantry‐mounted kV system used to acquire both 2D and 3D clinical images **must** have documented imaging doses for both 2D and 3D modes. If imaging dose is only measured for one acquisition technique, then the chosen technique **should** serve to provide the most conservative value (e.g., choose the acquisition technique that results in the greatest imaging dose). For 2D kV systems operated in fluoroscopy mode, the entrance exposure rate **should** be measured for the technique expected to produce the highest value.

These recommendations **must** also be augmented with procedures required by state regulations (such as measurement of X‐ray tube voltage accuracy, where applicable). Furthermore, IGRT systems with known recurring problems **should** be subjected to more frequent QA at the discretion of the QMP. Annual end‐to‐end tests are also an effective method of assessing overall IGRT system accuracy, but are not required in this report.

## 6. Conclusions

IGRT is a powerful and increasingly essential component of clinical radiation oncology practice. Proper use and quality assurance of clinical IGRT systems are of critical importance to maximizing the benefits and minimizing the risks of the technology. The minimum technical requirements for managing a clinical IGRT program stated in this document will help to achieve a more uniform standard of practice that improves the safety and quality of care of patients for whom IGRT is needed.

## ACKNOWLEDGMENTS

This guideline was developed by the Medical Physics Practice Guideline Task Group‐226 of the Professional Council of the AAPM.

TG‐226 MPPG 2.a. Members:

Jonas D. Fontenot, PhD, Chair

Hassaan Alkhatib, PhD

Jeffrey A. Garrett, MS

Andrew R Jensen, MS

Steven P. McCullough, PhD

Arthur J. Olch, PhD, FAAPM

Brent C. Parker, PhD

Ching‐Chong Jack Yang, PhD

Lynne A. Fairobent, AAPM Staff

AAPM Subcommittee on Practice Guidelines – AAPM Committee responsible for sponsoring the draft through the process:

Joann I Prisciandaro, PhD, Chair

Maria F. Chan, PhD, Vice‐Chair Therapy

Jessica B. Clements, MS

Dianna D. Cody, PhD, FAAPM,

Indra J. Das, PhD, FAAPM

Nicholas A. Detorie, PhD, FAAPM

Vladimir Feygelman, PhD

Luis E. Fong de los Santos, PhD

Jonas D Fontenot, PhD

David P Gierga, PhD

Kristina E. Huffman, MMSc

David W. Jordan, PhD

Ingrid R. Marshall, PhD

Arthur J. Olch, PhD, FAAPM

Robert J. Pizzutiello, Jr. MS, FAAPM, FACR, FACMP

Narayan Sahoo, PhD, FAAPM

J. Anthony Seibert, Chair, PhD, FAAPM, FACR, FSIIM

S. Jeff Shepard, MS, FAAPM, Vice‐Chair Imaging

Jennifer B. Smilowitz, PhD

James J. VanDamme, MS

Gerald A. White Jr., MS, FAAPM

Ning J. Yue, PhD, FAAPM

Lynne A. Fairobent, AAPM Staff

## References

[1] AAPM. Stereotactic body radiation therapy. AAMP TG‐101. Madison (WI): Medical Physics Publishing; 2010 Available from: http://www.aapm.org/pubs/reports/ 10.1118/1.3438081 20879569

[2] AAPM. The role of in‐room kV X‐ray imaging for patient setup and target localization. AAPM TG‐104. Madison (WI): Medical Physics Publishing; 2009 Available from: http://www.aapm.org/pubs/reports/

[3] AAPM. Quality assurance for image‐guided radiation therapy utilizing CT‐based technologies. AAPM TG‐179. Madison (WI): Medical Physics Publishing; 2012 Available from: http://www.aapm.org/pubs/reports/ 10.1118/1.3690466 22482616

[4] AAPM. The management of imaging dose during image‐guided radiotherapy. AAPM TG‐75. Madison (WI): Medical Physics Publishing; 2007 Available from: http://www.aapm.org/pubs/reports/ 10.1118/1.2775667 17985650

[5] AAPM. QA for helical tomotherapy. AAPM TG‐148. Madison (WI): Medical Physics Publishing; 2010 Available from: http://www.aapm.org/pubs/reports/ 10.1118/1.3462971 20964201

[6] AAPM. Clinical use of electronic portal imaging. AAPM TG‐58. Madison (WI): Medical Physics Publishing; 2001 Available from: http://www.aapm.org/pubs/reports/

[7] AAPM. Quality assurance for robotic radiosurgery. AAPM TG‐135. Madison (WI): Medical Physics Publishing; 2011 Available from: http://www.aapm.org/pubs/reports/ 10.1118/1.3579139 21815366

[8] AAPM. Quality assurance of medical accelerators. AAPM TG‐142. Madison (WI): Medical Physics Publishing; 2009 Available from: http://www.aapm.org/pubs/reports/

[9] AAPM. Comprehensive methodology for the evaluation of radiation dose in X‐ray computed tomography. AAPM TG‐111. Madison (WI): Medical Physics Publishing; 2010 http://www.aapm.org/pubs/reports/

[10] ACR‐ASTRO. Practice guideline for image‐guided radiation therapy (IGRT). Reston, VA: ACR; 2009. 10.1097/COC.0000000000000697 32452841

[11] AAMD. Scope of Practice for a Medical Dosimetrist. Herndon, VA: AAMD; 2012 Available from: http://www.medicaldosimetry.org/generalinformation/scope.cfm

[12] ASRT. The practice standards for medical imaging and radiation therapy: radiation therapy practice standards. Albuquerque, NM: ASRT; 2011 Available from: http://www.asrt.org/main/standards‐regulations/practice‐standards/practice‐standards

[13] AAPM. Statement on the description of involvement of Medical Physicists in clinical procedures. Professional Policy 18. College Park, MD: AAPM; 2007 Available from: http://www.aapm.org/org/policies/details.asp?id=238&type=PP

